# Liquiritin Attenuates Angiotensin II-Induced Cardiomyocyte Hypertrophy via ATE1/TAK1-JNK1/2 Pathway

**DOI:** 10.1155/2022/7861338

**Published:** 2022-03-16

**Authors:** Jiajia Mo, Peng Zhou, Zhaoxing Chu, Yan Zhao, Xiang Wang

**Affiliations:** ^1^Hefei Industrial Pharmaceutical Institute Co., Ltd., Hefei, Anhui 230051, China; ^2^Anhui Province Engineering Technology Research Center of Pharmaceutical Re-innovation, Hefei, Anhui 230051, China; ^3^Anhui University of Chinese Medicine, Hefei, Anhui 230012, China

## Abstract

**Objective:**

To investigate the protective effect and mechanism of liquiritin (LIQ) on cardiomyocyte hypertrophy induced by angiotensin II (Ang II).

**Methods:**

H9c2 cells were pretreated with LIQ before and after Ang II treatment. CCK8 assay was performed to evaluate cell viability. The cell surface area was measured by phalloidin staining. The mRNA expression of atrial and B-type natriuretic peptides (ANP and BNP, respectively) and *β*-myosin heavy chain (*β*-MHC) was determined by quantitative reverse transcription-polymerase chain reaction (RT-qPCR); the protein levels of arginyltransferase 1 (ATE1), transforming growth factor beta-activated kinase 1 (TAK1), phos-TAK1, c-Jun N-terminal kinases1/2 (JNK1/2), and phos-JNK1/2 were determined by Western blotting. After constructing the ATE1 overexpression cell models with the pcDNA3.1/ATE1, the abovementioned indicators were tested using the introduced methods.

**Results:**

LIQ at a concentration of ≤30 *μ*M was not cytotoxic to H9c2 cells before exposure to Ang II. The protective effect of LIQ was best observed at 30 *μ*M after Ang II treatment. Phalloidin staining and RT-qPCR results indicated that the deposition of Ang II increased the cell surface area and levels of ANP, BNP, and *β*-MHC. On the other hand, Western blotting results showed that Ang II increased the ATE1 protein levels and TAK1 and JNK1/2 phosphorylation, which were significantly alleviated after LIQ treatment. LIQ also directly inhibited the ATE1 overexpression in H9c2 cells transfected with pcDNA3.1/ATE1 and further inhibited TAK1 and JNK1/2 phosphorylation.

**Conclusion:**

LIQ can attenuate Ang II-induced cardiomyocyte hypertrophy by regulating the ATE1/TAK1-JNK1/2 pathway.

## 1. Introduction 

Heart failure is a common life-threatening medical condition with high morbidity and mortality globally [1]. Cardiac hypertrophy, which is accompanied by increased cell death, fibrotic remodeling, and contractile dysfunction, is considered to play a pivotal role in the pathophysiological processes of heart failure [2, 3]. Hence, inhibiting or delaying the progression of pathological cardiac hypertrophy can serve as an effective therapeutic strategy for preventing heart failure [4]. Currently, no effective drug is available to prevent or treat cardiac hypertrophy, and hence, developing appropriate novel medications is crucial.

Previous studies have reported that several factors such as inflammation, oxidative stress, apoptosis, and autophagy are associated with cardiac hypertrophy [5, 6]. Furthermore, multiple signaling pathways are involved in cardiac hypertrophy development, among which ATE1 is an important target and acts as a regulator of hypertrophic response [7, 8]. A recent study showed that ATE1 regulates the expression of hypertrophic gene markers via the TAK1-JNK1/2 signaling pathway, which provides a potentially novel therapeutic target for treating cardiac hypertrophy [9].

Licorice (*Glycyrrhiza radix*), a popular herb in traditional Chinese medicine (TCM), has been used to treat cardiovascular diseases for thousands of years and has been reported to exhibit anticardiac hypertrophy activity [10–12]. LIQ, the main flavone compound derived from licorice, exhibits anti-inflammatory, antioxidant, antiapoptotic, and cardioprotective activities [13–16]. Previous studies indicated that LIQ could alleviate myocardial fibrosis and attenuate myocardial damage [17, 18]. Although licorice exhibits antihypertrophic activity, the antihypertrophic effect of its main component LIQ and the mechanism underlying the same remain unclear. Therefore, we aimed to explore the antihypertrophic effects of LIQ on Ang II-induced cardiomyocyte hypertrophy and whether its possible underlying mechanism involves the ATE1/TAK1-JNK1/2 pathway.

## 2. Materials and Methods

### 2.1. Chemicals and Reagents

LIQ (CAS No. 551-15-5; purity ≥ 98%), Ang II was sourced from Shanghai YuanYe Biotechnology (Shanghai, China). Primary antibodies including anti-TAK1, anti-phos-JNK1/2, and anti-JNK1/2 were purchased from CST (Boston, USA). Primary antibodies including anti-ATE1 and secondary antibodies were purchased from Abcam (Cambridge, England); anti-phos-TAK1 was purchased from Sabbiotech (Maryland, USA). CCK-8 was purchased from Beyotime Biotechnology (Shanghai, China). The Phalloidin Staining Kit was purchased from Solarbio (Beijing, China). The RT-qPCR kit, Trizol, and the SYBR Premix Ex Tap kit were purchased from Takara (Japan). Real-time PCR primers were obtained from Sangon Biotech (Shanghai, China). All other chemicals used were sourced from Thermo Fisher Scientific (Pittsburgh, PA, USA).

### 2.2. Cell Culture and Treatment

H9c2 rat cardiomyocyte cells, purchased from the American Type Culture Collection (Rockville, MD, USA), were cultured in DMEM containing 10% fetal bovine serum, 100 U/mL penicillin, and 100 *µ*g/mL streptomycin at 37°C under a 5% CO_2_ incubator. The cells in the logarithmic growth phase were selected for the experimental design. The concentrations of LIQ were selected based on the cell viability. After 24 h of culture, the H9c2 cells were then assigned to three groups: (1) control group: the cells were cultured normally; (2) Ang II group: the cells were treated with 1 *μ*M Ang II for 24 h; and (3) LIQ group: the cells were treated with 30 *μ*M LIQ for 1 h before Ang II administration. Each group was incubated in an incubator with 5% CO_2_ at 37°C [9, 19]. Ang II was dissolved in PBS, and LIQ was dissolved in DMSO, where the final concentration of DMSO was <0.1%. To further evaluate the protective effect of LIQ on rat cardiomyocytes through the regulation of the ATE1/TAK1-JNK1/2 pathway, we overexpressed ATE1 with ATE1 plasmids on the basis of LIQ and observed its protective effect on the H9c2 cells again.

### 2.3. Cell Viability Assay

The H9c2 cells were cultured in 96-well plates at a density of 5 × 10^3^ cells per well. To test the cell viability under LIQ treatment, the cells were treated with various concentrations of LIQ and incubated for 24 h. To evaluate the cell viability under Ang II and LIQ treatment, the cells were administered with Ang II, LIQ, or a combination of the two drugs. Then, the number of living cells was enumerated by the CCK8 assay. Briefly, the cells were incubated in serum-free RPMI-1640 medium for 2 h, after which the supernatants were discarded. Then, 10 *μ*L of CCK8 was added to each well at the end of drug treatment. After 4 h of incubation at 37°C, the number of surviving cells and the half-maximal inhibitory concentrations (IC_50_) value of LIQ were analyzed by detecting the absorbance at 450 nm on a microplate reader.

### 2.4. Plasmid Transfection

The pcDNA3.1/ATE1 plasmid and the vector used in this study were provided by GenePharma (Shanghai, China). At 24 h before the transfection, the H9c2 cells were digested in the logarithmic growth phase with trypsin, and the cell density was adjusted to 5 × 10^6^ cell/15 mL with 10% fetal bovine serum, followed by seeding on 10 cm dishes in the complete medium. The cells were then used for transfection when the density reached 70–80%. At 2 h before transfection, the cell medium was changed to a serum-free medium. Then, the plasmid was transfected into cells with the Lipofectamine 2000 (Thermo Fisher, NY, USA) following the supplier's instructions. Briefly, 3.2 *μ*g of plasmid was mixed with 200 *μ*L of Opti-MEM, 8 *μ*L Lipofectamine 2000 was mixed with 200 *μ*L Opti-MEM, and the transfection mix was prepared by mixing the plasmid preparation solution with an equal volume of the Lipofectamine 2000 solution. After incubating at room temperature for 30 min, the transfection mix solution was added to the cell culture plates and mixed gently. Subsequently, the cells were incubated for 6 h at 37°C in a 5% CO_2_ humidified atmosphere. The transfection medium was then substituted with the complete medium.

### 2.5. RT-qPCR Analysis

Cell suspensions from different groups were prepared and seeded into a 6-well plate at the density of 1 × 10^6^ cells/well. Total RNA was extracted from each group of the H9c2 cells by the Trizol method as described by the manufacturer. RNA was reverse transcribed into cDNA using the cDNA Synthesis Kit. The real-time reaction was conducted with the ABI 7500 Real-Time PCR system. The relative expressions of ANP, BNP, *β*-MHC, and ATE1 mRNA levels were calculated based on the 2^−ΔΔ*Ct*^ method. The specific primers sequences (both forward and reverse) used in this study are shown in Table 1.

### 2.6. Measurement of Cell Surface Area

The cell surface area was observed by phalloidin staining. Briefly, following drug treatment or transfection, the H9c2 cells were washed thrice with PBS, fixed with 3.75% formaldehyde, and then incubated on an ice bath for 15 min, after which the cells were permeabilized with 0.5% Triton X-100 for 10 min. After washing thrice with PBS, the diluted phalloidin solution (5 *μ*g/mL) was added dropwise and the cells were incubated for 20 min at room temperature. The cells were then washed again with PBS thrice and incubated with DAPI in the dark for 5 min. Subsequently, excess DAPI was removed with PBS wash, and the cells were observed and photographed by the ZEISS LSM Laser Confocal Microscope system.

### 2.7. Western Blotting Analysis

Total proteins were extracted from the H9c2 cells in different groups by using lysis buffer and quantified by the BCA assay. Then, the proteins were loaded into the SDS-polyacrylamide gel and separated by electrophoresis. After electrophoresis, these separated proteins were then transferred onto PVDF membranes, which were then cut according to the molecular weight of the target protein. After blocking with 5% skim milk in TBST for 1 h, the membranes were incubated under 4°C overnight with primary antibodies (Table 2). On the following day, the membranes were washed with TBST and incubated with the corresponding secondary antibodies at room temperature for 1 h. The resultant protein bands were visualized using the Tanon 6600 Luminescent Imaging Workstation (Tanon, China), and the relative intensity was measured and analyzed by using the Image-Pro Plus 6.0 software.

### 2.8. Statistical Analysis

All data were expressed as means ± SD. The statistical analysis was performed using the statistical software SPSS23.0 or Graphpad 6.0 software. One-way analysis of variance (ANOVA) and Tukey's test were performed to evaluate the differences between the groups. *P* < 0.05 was considered to indicate significant differences.

## 3. Results

### 3.1. Effect of LIQ on the Viability of H9c2 Cells

**Figure 1:**
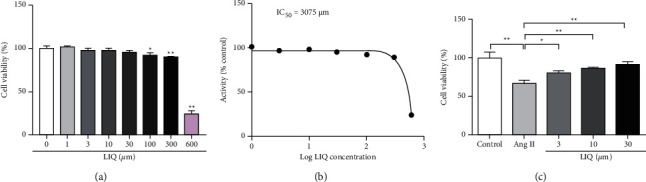
Effect of LIQ on the viability of H9c2 cardiomyocytes. (a) Effects of different concentrations of LIQ on the survival rate of normal H9c2 cells assessed by the CCK8 assay. (b) The IC_50_ value of LIQ in H9c2 cells was analyzed by the CCK8 assay. (c) The H9c2 cells were pretreated for 1 h with LIQ (3 *μ*M, 10 *μ*M, 30 *μ*M), Ang II (1 *μ*M) was then added, and the cells were incubated for 24 h. The cell viability was measured with a CCK8 assay. The results were expressed as mean ± SD (*n* = 3). ^*∗*^*P* < 0.05,^∗∗^*P* < 0.01.

To determine whether LIQ exhibits any cytotoxic effect, H9c2 cells were cultured in the presence of different concentrations of LIQ (1, 3, 10, 30, 100, 300, and 600 *μ*M) for 24 h. CCK-8 assays were performed to determine cell viability. The results indicated that LIQ at concentrations of 30 *μ*M or lower was not cytotoxic to H9c2 cells, whereas, at concentrations of 100 *μ*M or higher, it significantly promoted cytotoxicity in H9c2 cells (*P* < 0.05, *P* < 0.01) and the IC_50_ was 3075 *μ*M (Figures 1(a), 1(b)). Therefore, LIQ at concentrations of 3, 10, and 30 *μ*M were selected to confirm its effect on the viability of H9c2 cardiomyocytes after Ang II (1 *μ*M) treatment. As shown in Figure 1(c), the cell viability of the Ang II group decreased significantly compared to that of the control group (*P* < 0.01). Treatment with LIQ at concentrations of 3 *μ*M, 10 *μ*M, and 30 *μ*M increased cell viability compared to the Ang II group (*P* < 0.05, *P* < 0.01). The effect of LIQ was best recorded at 30 *μ*M, which is a concentration used for investigating its antihypertrophic effect.

### 3.2. Antihypertrophic Effect of LIQ on Ang II-Treated H9c2 Cells

**Figure 2:**
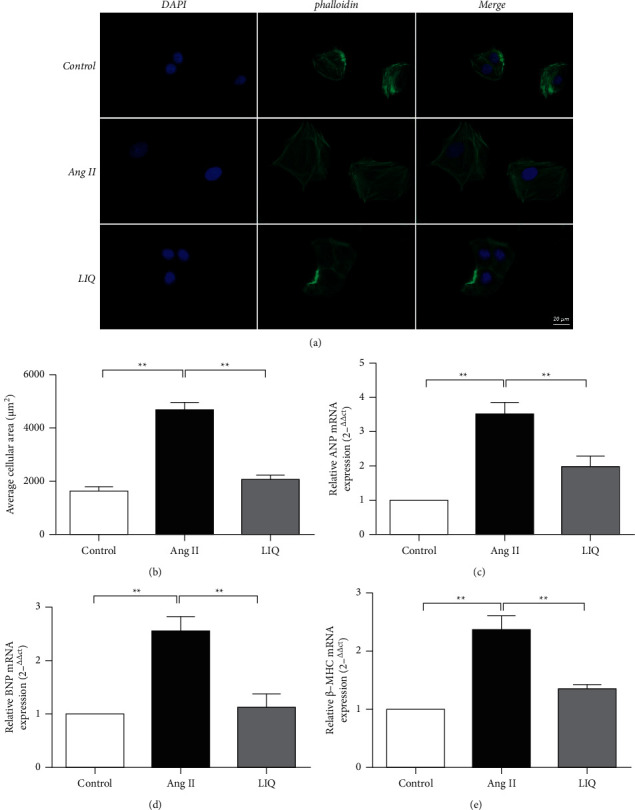
Antihypertrophic effect of LIQ on Ang II-treated H9c2 cells. The cell surface area was evaluated by phalloidin staining and observed by fluorescence microscopy. Tetramethylrhodamine B isothiocyanate-labeled ghost cyclic peptide staining was used to stain the cytoskeleton (green) and the nucleus (blue) was stained by DAPI staining (a, b). The mRNA levels of ANP, BNP, and *β*-MHC were detected by RT-qPCR. The mRNA levels of ANP (c), BNP (d), and *β*-MHC (e) were normalized to those of the control. The results were expressed as mean ± SD (*n* = 3). ^*∗*^*P* < 0.05,^∗∗^*P* < 0.01.

Cardiomyocyte hypertrophy response is characterized by augmented cell size and the induced expression of hypertrophic markers such as ANP, BNP, and *β*-MHC [20]. Therefore, cell surface area and the mRNA levels of classical markers of hypertrophy were determined in our study. As shown in Figure 2, the cell surface areas of cardiomyocytes (Figures 2(a) and 2(b)) and the levels of ANP, BNP, and *β*-MHC (Figures 2(c)–2(e)) markedly increased in the Ang II-treated group (*P* < 0.01), indicating the occurrence of hypertrophy in these cells. However, a reduction in Ang II-induced cardiomyocyte enlargement was observed in the LIQ-pretreated group compared with that in the Ang II-treated group (*P* < 0.01), which was accompanied by a significant decrease in the ANP, BNP, and *β*-MHC levels (*P* < 0.01). These findings demonstrate that LIQ could inhibit cardiac hypertrophy in vitro.

### 3.3. LIQ Regulated the Expression of ATE1, TAK1, and JNK1/2 in Ang II-Treated H9c2 Cells

**Figure 3:**
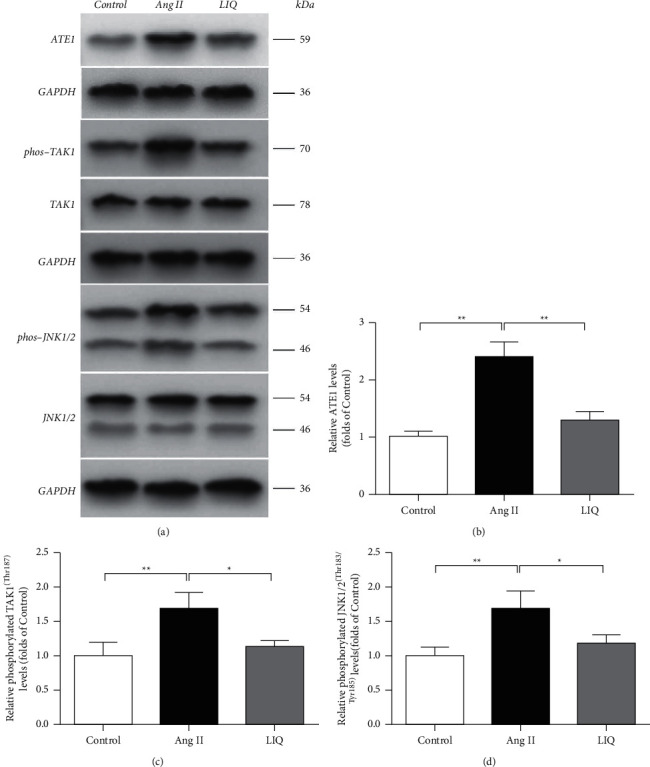
Effect of LIQ on the ATE1/TAK1-JNK1/2 pathway in Ang II-treated H9c2 cells. The expressions of ATE1, TAK1, phos-TAK1, JNK1/2, and phos-JNK1/2 from the indicated group were detected by Western blotting; the representative bands were shown in panel (a). The levels of ATE1 (b), phos-TAK1 (c), and phos-JNK1/2 (d) were normalized to those of the control. The results were expressed as mean ± SD (*n* = 3). ^*∗*^*P* < 0.05,^∗∗^*P* < 0.01.

To determine the regulatory mechanism by which LIQ inhibits cardiac hypertrophy, we tested the ATE1 signaling pathway. Western blotting results indicated that the protein levels of ATE1 and the phosphorylation of TAK1 (Thr187) and JNK1/2 (Thr183/Tyr185) in the Ang II group significantly increased than those in the control group (*P* < 0.01). Compared with the Ang II group, LIQ pretreatment considerably downregulated the phosphorylation of TAK1 and JNK1/2 and ATE1 expression (*P* < 0.05, *P* < 0.01) (Figure 3).

### 3.4. Overexpression of ATE1 by Plasmid Transfection

**Figure 4:**
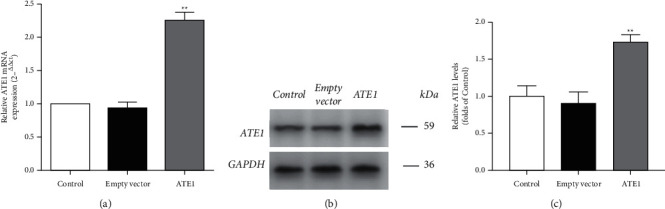
The effect of mRNA and protein expressions of ATE1 in the H9c2 cells. The mRNA and protein expression levels of ATE1 were detected by RT-qPCR analysis (a) and Western blotting (b, c). The results were expressed as mean ± SD (*n* = 3). ^∗∗^*P* < 0.01 vs. empty vector.

To further delineate the mechanisms underlying the inhibitory effect of LIQ against cardiac hypertrophy, H9c2 cells were transfected with an ATE1 overexpression plasmid. An empty vector plasmid was used as a negative control. The transfection efficiency of the plasmid vectors was evaluated by real-time PCR and Western Blotting. As shown in Figure 4, the levels of ATE1 mRNA and protein expression in the ATE1 group increased significantly compared with those in the empty vector group (*P* < 0.01). This result suggested that ATE1 was successfully overexpressed in H9c2 cells by transfection.

### 3.5. Effect of LIQ on Cell Size and the Levels of ANP, BNP, and *β*-MHC in Ang II-Treated H9c2 Cells after ATE1 Overexpression

**Figure 5:**
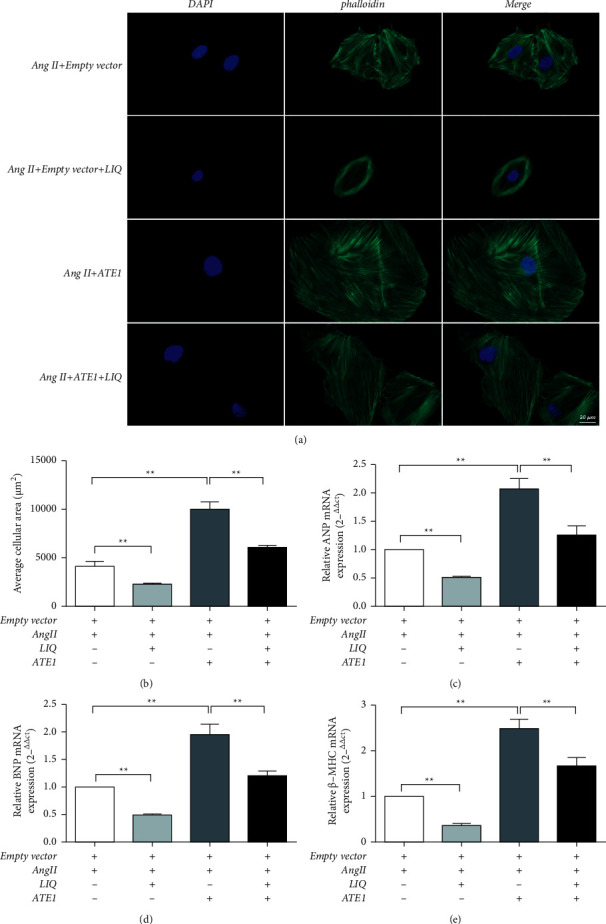
LIQ inhibits the cell size and the levels of ANP, BNP, and *β*-MHC in Ang II-treated H9c2 cells upon overexpression of ATE1. The cell surface area was observed by phalloidin staining and then fluorescence microscopy. Tetramethylrhodamine B isothiocyanate-labeled ghost cyclic peptide staining was performed to stain the cytoskeleton (green) and the nucleus (blue) was stained by DAPI staining (a, b). The mRNA levels of ANP, BNP, and *β*-MHC were detected by RT-qPCR. The mRNA levels of ANP (c), BNP (d), and *β*-MHC (e) were normalized to those of the control. The results were expressed as mean ± SD (*n* = 3). ^*∗*^*P* < 0.05,^∗∗^*P* < 0.01.

As shown in Figure 5, the cell surface areas of cardiomyocytes (Figures 5(a) and 5(b)) and the levels of ANP, BNP, and *β*-MHC (Figures 5(c)–5(e)) markedly increased after ATE1 overexpression (*P* < 0.01). These results suggested that ATE1 overexpression in H9c2 cells promoted hypertrophy induced by Ang II. These results were in line with the results reported previously [9]. After LIQ intervention, the cell surface area and the levels of ANP, BNP, and *β*-MHC decreased significantly (*P* < 0.01). Thus, ATE1 can be considered a potential target when using LIQ for treating cardiac hypertrophy.

### 3.6. Effect of LIQ on the ATE1/TAK1-JNK1/2 Pathway in Ang II-Treated H9c2 Cells after ATE1 Overexpression

**Figure 6:**
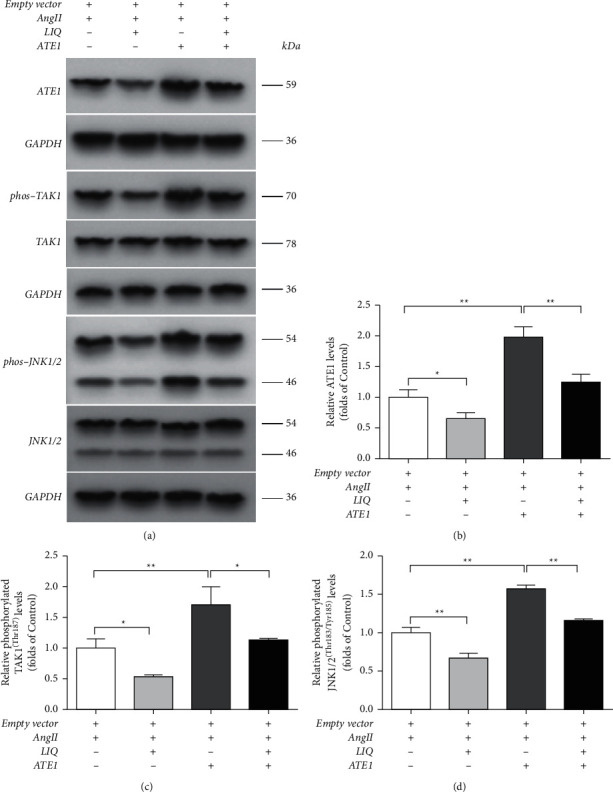
LIQ inhibited the TAK1-signaling pathway in Ang II-treated H9c2 cells upon overexpression of ATE1. The expressions of ATE1, TAK1, phos-TAK1, JNK1/2, and phos-JNK1/2 from the indicated group were detected by Western blotting; the representative bands were shown in panel (a). The levels of ATE1 (b), phos-TAK1 (c), and phos-JNK1/2 (d) were normalized to those of the control. The results were presented as mean ± SD (*n* = 3). ^*∗*^*P* < 0.05,^∗∗^*P* < 0.01.

As shown in Figure 6, the ATE1 protein levels and the phosphorylation of TAK1 (Thr187) and JNK1/2 (Thr183/Tyr185) significantly increased after ATE1 overexpression (*P* < 0.01). After LIQ treatment, the phosphorylation of TAK1 and JNK1/2 and ATE1 expression considerably reduced (*P* < 0.05, *P* < 0.01). Taken together, these results demonstrate that LIQ could inhibit Ang II-induced cardiac hypertrophy by inhibiting the ATE1/TAK1-JNK1/2 pathway.

## 4. Discussion

Cardiac hypertrophy, characterized by an increase in cardiomyocyte size, enhanced fetal gene expression, accelerated interstitial cell proliferation, and a higher organization of the sarcomeric structure, is a compensatory mechanism in response to a variety of mechanical and neurohormonal stimuli such as Ang II, ET-1, catecholamines, and adrenaline [21, 22]. Although this process is initially an adaptive response to meet the increased demand, persistent hypertrophy frequently induces arrhythmia and can cause heart failure and sudden cardiac death [23]. Among all the stimuli that contribute to the development of cardiac hypertrophy, Ang II has been confirmed as a classic factor for the initiation of cardiac hypertrophy [24]. It can directly regulate myocardial contractility, energy metabolism, and hypertrophic growth [25, 26]. Therefore, the inhibition of Ang II-induced cardiomyocyte hypertrophy has been regarded as a key strategy for preventing cardiac diseases. In the present study, H9c2 cells treated with Ang II were used to simulate cardiac hypertrophy, which is the mechanism mainly induced by stress [27]. In the current experiments, our results revealed that untreated H9c2 cells grew well, whereas significant enlargement in the size of cardiomyocytes was observed after treatment with Ang II (Figures 2(a) and 2(b)). While treatment of these cells with Ang II induced an obvious increase in ANP, BNP, and *β*-MHC levels (Figures 2(c)–2(e)), the levels of these hypertrophic markers have also been found to be increased in the hearts of patients with myocardial hypertrophy [28, 29]. LIQ pretreatment could improve morphological changes and decrease ANP, BNP, and *β*-MHC gene expression by regulating the ATE1/TAK1-JNK1/2 signaling pathway. These results indicate that LIQ can inhibit cardiac hypertrophy induced by Ang II, thereby providing new insights into its therapeutic effects.

Licorice is a commonly used TCM and is frequently ranked in the top 5 in TCM treatment of chronic heart failure [30]. LIQ is one of the main active ingredients of licorice, and past studies have demonstrated the cardiovascular protective effect of LIQ from diverse perspectives. For example, LIQ has been demonstrated to protect from high fructose-induced myocardial fibrosis, possibly through the inhibition of the NF-*κ*B and MAPKs signaling pathways [18]. Yi et al. [31] demonstrated that the protective effects of LIQ on myocardial tissue in aconitine induced cardiotoxicity occurred by synergistically inhibiting CaM expression and Cx43 dephosphorylation. Another study indicated that LIQ exerts cardioprotective effects by reducing oxidative stress, inflammation, apoptosis, and metabolic alteration by regulating the AMPK*α*2-dependent signaling pathway in LPS induced cardiomyocyte injury [17]. In addition, Vu et al. [32] confirmed that LIQ could inhibit hypoxia/reoxygenation- (HR-) induced H9c2 cell death by preserving mitochondria and elevating the mitochondrial Ca^2+^ level. These studies suggest that LIQ can play a cardioprotective role by regulating the stress response. Similarly, our results also indicated for the first time that LIQ treatment could prevent Ang II-induced cardiomyocyte stress injury and increased cell viability (Figure 1(c)).

ATE1 is a highly functionally conserved enzyme present in all eukaryotes [33]. It is the only known enzyme in metazoans and fungi that is capable of catalyzing posttranslational arginylation. Arginylation is one of the major pathways of protein posttranslational modification. Previous studies have demonstrated that ATE1-mediated arginylation is the link between the ubiquitin-dependent degradation of a protein and the identity of its N-terminal amino acid residue, which subsequently regulates embryonic development, protein synthesis, nuclear activity, aging, etc. [33, 34]. Therefore, ATE1 is the primary regulator of eukaryotic cellular homeostasis. Impairment of ATE1 function has been associated with a variety of human disorders such as congenital heart defects, cancer, neurodegeneration, and obesity, wherein changes in the response to different types of stress, regulating metabolism, and apoptosis have been observed, which eventually affect mitochondrial function [35–40]. The relationship between ATE1 function and heart diseases has been demonstrated in recent years. A previous study showed that deleting the ATE1 gene caused embryonic lethality in mice during the mid-gestational stage, with severe defects in cardiac morphogenesis and angiogenesis [7]. Also, knocking out of the ATE1 gene could induce autonomous changes in cardiac myocytes and disrupt myocardial integrity and myocyte contractility at different embryonic stages [8]. Moreover, the absence of ATE1 in the heart muscles has been reported to cause progressive dilated cardiomyopathy and a spectrum of heart abnormalities in mice [35, 41]. These results partly explain how ATE1 gene deletion affects the phenotype of a normal embryonic heart. However, in response to increased cardiac stress, the expression of the ATE1 gene and protein in adult rats was strongly upregulated, and the loss of ATE1 gene expression in this type of cardiac cells suppressed the expression of genes that regulate cardiac hypertrophy [9]; this effect may be potentially related to downregulation of arginylation after ATE1 knockout or silencing, which makes the cells less sensitive to different stress factors [39]. Increasing evidence suggests that arginylation occurs as a response to a variety of stress, preferentially on damaged proteins [42, 43]. This result indicates that ATE1 gene knockdown under the condition of cardiac stress can confer cardioprotection, whereas the inhibition of the ATE1 may lead to therapeutic intervention for preventing myocardial hypertrophy. Existing evidence suggests that ATE1 is involved in Ang II stress-induced cardiac hypertrophy in H9c2 cells, while LIQ has obvious antioxidative stress and cardioprotective effects. Hence, it can be reasonably inferred that the inhibitory effect of LIQ on cardiac hypertrophy may be partly dependent on stress-induced cardiac hypertrophy mediated through ATE1 activation. The results of our study confirmed this hypothesis as we discovered that LIQ significantly inhibited Ang II-induced surface area increase of H9c2 cells and decreased the expression of cardiac hypertrophy markers ANP, BNP, and *β*-MHC mRNA in vitro by downregulating the ATE1 protein expression (Figures 2(a)–2(e) and 3(a) and 3(b)). To confirm these observations, we overexpressed ATE1 in H9c2 cells and repeated the experiments. Interestingly, LIQ also inhibited the ATE1 protein overexpression and surface area increase of H9c2 cells (Figures 5(a) and 5(b) and 6(a) and 6(b)), which confirmed that ATE1 may function as a key protein and potential target for LIQ to exert an anticardiac hypertrophy effect.

TAK1 belongs to the mitogen-activated protein kinase kinase kinase family produced in response to transforming growth factor-*β* [44], which is expressed in cardiomyocytes [45]. Under physiological conditions, sufficient levels of TAK1 are indispensable for cardiomyocyte survival [46]; however, some previous studies proved that myocardial TAK1 elicited chamber enlargement and heart remodeling [47, 48]. TAK1 level was reported to be markedly increased in the mouse heart under pathological stress such as pressure overload and myocardial infarction, as well as in the human myocardium [45, 49]. Further, TAK1 activation exacerbated hypertrophic growth in the presence of pressure overload or neurohormonal stimuli, whereas the cardiac-specific overexpression of active TAK1 was sufficient to cause myocardial hypertrophy and dysfunction [50, 51]. Upon stimulation, TAK1 is activated and then phosphorylated by binding to the polyubiquitin chain, and phosphorylated TAK1 can transduce the signal to the downstream kinases JNK1/2, which are also phosphorylated and activated [52]. JNK1/2, which can phosphorylate the N-terminal of the transcription factor c-Jun in response to a variety of stresses, are involved in many physiological and pathological processes. The pharmacological blockage of JNK1/2 could delay the transition toward heart failure, whereas their overactivation can induce cardiac dilation and dysfunction [53, 54]. Growing evidence has shown that the TAK1-JNK1/2 signaling is strongly implicated in controlling the expression of hypertrophic markers and cell size [50, 55]. In our study, we observed that TAK1 and JNK are involved in the process of LIQ inhibiting cardiac hypertrophy. We have revealed, for the first time, through this study that LIQ inhibits cardiac hypertrophy by downregulating the expression of ATE1 and the phosphorylation of TAK1 and JNK1/2 (Figures 3(a)–3(d)), which inhibits the mRNA expression of hypertrophy-related markers ANP, BNP, and *β*-MHC (Figures 2(a)–2(e)). By overexpressing ATE1 in H9c2 cells through plasmid transfection and repeated experiments, we found that the mRNA expression levels of ANP, BNP, and *β*-MHC and phosphorylation levels of TAK1 and JNK1/2 were inhibited after LIQ treatment (Figures 5(c)–5(e) and 6(c) and 6(d)). Accordingly, LIQ inhibited H9c2 cardiomyocyte hypertrophy induced by Ang II through the ATE1/TAK1-JNK1/2 signaling pathway.

At present, there are few studies on the ATE1 signaling pathway. The highlight of this study is the first to discover that LIQ can inhibit Ang II-induced cardiac hypertrophy by regulating the expression of ATE1 and thereby downregulating the ATE1/TAK1-JNK1/2 signaling pathway. However, it is not yet clear how LIQ regulates the ATE1 expression. Mitochondria are the main source of oxidative stressors in cells, and studies have shown that the expression of ATE1 increases in proportion to the degree of various stresses [39]. A large number of genes (at least 19) related to mitochondria or energy production, such as Oma1 and COQ7, were found to have genetic interactions with ATE1 through GO term studies, and those partial proteins have been reported to be involved in the regulation of cardiac hypertrophy [56–58]. Future studies would be required to study which proteins/factors are involved in LIQ downregulating ATE1 expression and its relationship with anticardiac hypertrophy from the stress response.

## 5. Conclusion

**Figure 7:**
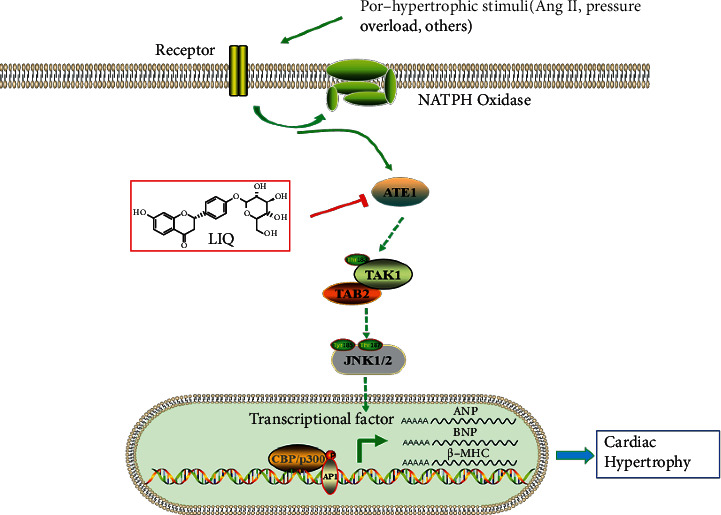
Schematic diagram depicting the effects of LIQ on cardiac hypertrophy via inhibiting the ATE1/TAK1-JNK1/2 signaling pathway.

In the present study, we have clearly demonstrated a previously unidentified effect of LIQ on Ang II-induced cardiac hypertrophy. We also confirmed that the protective effect of LIQ was achieved through the suppression of the ATE1/TAK1-JNK1/2 pathway (Figure 7). Our results thus provide the necessary experimental evidence asserting that LIQ is a potential therapeutic candidate for the treatment of cardiac hypertrophy and heart failure.

## Figures and Tables

**Table 1:** Primers used in Quantitative Real-Time PCR.

Primers		Sequence (5′⟶3′)
ANP	Forward	5′-CCTGGACTGGGGAAGTCAAC-3′
	Reverse	5′-ATCTATCGGAGGGGTCCCAG-3′

BNP	Forward	5′-TTAGGTCTCAAGACAGCGCC-3′
	Reverse	5′-CGCCGATCCGGTCTATCTTC-3′

*β*-MHC	Forward	5′-AAGGCCAAGATCGTGTCTCGAGA-3′
	Reverse	5′-ACACAGAAGAGGCCTGAGTAGGTG-3′

ATE1	Forward	5′-CAACTGTGAGCCAGGCAGAG-3′
	Reverse	5′-CACCCCCACAGCAATGATCT-3′

GAPDH	Forward	5′- AAGAGGGATGCTGCCCTTAC -3′
	Reverse	5′- ATCCGTTCACACCGACCTTC-3′

**Table 2:** Antibodies used in the study.

Antibody	Suppliers	Cat. no.	Dilution
ATE1	Abcam	ab199423	1 : 1000
TAK1	CST	5206	1 : 1000
Phos-TAK1	Sabbiotech	12255	1 : 2000
Phos-JNK1/2	CST	4668	1 : 1000
JNK1/2	CST	9252	1 : 1000
Goat anti-rabbit IgG H&L (HRP)	Abcam	ab205718	1 : 10000
Anti-rabbit IgG H&L (alexa Fluor® 488)	Abcam	ab150077	1 : 10000

## Data Availability

The data used to support the findings of this study are available upon request to the corresponding author.
